# Pembrolizumab-Induced Diabetic Ketoacidosis: A Review of Critical Care Case

**DOI:** 10.7759/cureus.18983

**Published:** 2021-10-22

**Authors:** Keerthana Sankar, Matthew Macfarlane, Odelia Cooper, Jeremy Falk

**Affiliations:** 1 Internal Medicine, Cedars-Sinai Medical Center, Los Angeles, USA; 2 Endocrinology and Diabetes, Cedars-Sinai Medical Center, Los Angeles, USA; 3 Pulmonary and Critical Care Medicine, Cedars-Sinai Medical Center, Los Angeles, USA

**Keywords:** immune-related adverse event, pd-1 inhibitor, diabetes, immunotherapy, immune checkpoint inhibitors, diabetic ketoacidosis

## Abstract

Diabetic ketoacidosis (DKA) is a commonly encountered diagnosis in the general inpatient and intensive care unit settings. We report a rare case of pembrolizumab-induced DKA in a patient with bladder carcinoma in situ with no prior diagnosis of diabetes. Our case highlights the importance of understanding immune-related adverse events (IRAEs) as immunotherapy is becoming a mainstay of treatment for a variety of diagnoses. The rare side effect of DKA presented in this case is compared to the classical presentation of DKA secondary to type 1 diabetes mellitus (T1DM). We found that pembrolizumab-induced DKA presented with fewer symptoms than T1DM-induced DKA and did not present with serum antibodies that are typically present in T1DM. While management of DKA in the acute setting is unchanged regardless of the precipitating factor, this case demonstrates the importance of identifying the precipitant in order to pursue the appropriate diagnostic workup and long-term management.

## Introduction

Immune checkpoint inhibitors (ICIs) are targeted, cell-directed therapies that are becoming more common in the treatment of various cancers. The rate of fatal immune-related adverse events (IRAEs) ranges from 0.3% to 1.3% which is substantially lower than traditional systemic chemotherapeutic agents [1]. However, the long-term side effects of ICIs have yet to be studied extensively. Though many systemic chemotherapeutic agents have been in use for decades, adverse reactions to these agents continue to be discovered more than three decades after its initial use [2]. This demonstrates the need to continue elucidating the side effect profile of newer medications. Pembrolizumab is approved in the United States for the treatment of melanoma, head and neck cancers, certain lymphomas, and urothelial carcinoma. Current data shows that IRAEs, specifically those related to anti-programmed death 1 (PD1) medications such as pembrolizumab, are most commonly associated with dermatologic disorders, pneumonitis, musculoskeletal disorders, and endocrine disorders [3]. We present a rare case of pembrolizumab-induced diabetic ketoacidosis (DKA) in a patient with bladder carcinoma in situ with no prior diagnosis of diabetes.

## Case presentation

An 85-year-old woman presented to the emergency room with acute onset lightheadedness, palpitations, and near syncope. She did not report nausea, vomiting, chest pain, abdominal pain, fevers, chills, polyuria, constipation, or diarrhea. The patient had a medical history of recurrent bladder carcinoma in situ which was originally diagnosed in 2013 and treated with intravesicular bacillus Calmette-Guerin (BCG). She had recurrence of cancer in 2018 and 2019 and was subsequently started on pembrolizumab nine months prior to her current presentation. She had no prior diagnosis of diabetes or history of gestational diabetes. Her home medication list is notable for simvastatin, pembrolizumab, omeprazole, and solifenacin. She did not report taking glucocorticoids. Social history did not reveal alcohol, tobacco, or drug use. Family history was unremarkable for endocrinopathies. Physical examination findings were notable for fatigued appearance (BMI 20.25 kg/m^2^) and a benign abdominal examination. Initial lab values showed an elevated blood glucose level five times the upper limit of normal, mild hyponatremia, elevated anion gap, elevated beta-hydroxybutyrate, and low serum bicarbonate, all of which indicated an anion gap metabolic acidosis consistent with DKA. Urinalysis was also notable for elevated glucose and ketones further supporting the diagnosis (lab values are reported in Table 1). Imaging studies of the pancreas were not performed. The patient was admitted to the intensive care unit for management of DKA.

**Table 1:** Pertinent Lab Values IGF-1: insulin-like growth factor 1 (secreted in response to growth hormone stimulation and has an anabolic effect on muscles, bones, and organs); T3: triiodothyronine (produced by the deiodination of free thyroxine and is a potent activator of cellular metabolism); LH: luteinizing hormone (secreted by the anterior pituitary gland and is utilized for ovarian follicle stimulation in females and androgen production in males)

Lab Values	Patient Results	Reference Range
Glucose (mg/dL)	555	70-99
Sodium (mmol/L)	133	135-145
Chloride (mmol/L)	104	98-107
Potassium (mmol/L)	4	3.5-5
Bicarbonate (mmol/L)	11	22-31
Anion Gap (meq/L)	18	8-12
Beta-Hydroxybutyrate (mmol/L)	4.9	<0.6
Hemoglobin A1c (%)	6.8	<5.7
IGF-1 (ng/mL)	18	34-246
T3 (ng/dL)	53	76-181
LH (MIU/mL)	14.3	15-62 (Post-Menopausal)
Urine Glucose (Qualitative)	4 +	Negative
Urine Ketones (Qualitative)	3 +	Negative

The patient was volume resuscitated, started on an insulin drip, and appropriately transitioned to subcutaneous insulin. A limited autoimmune hypophysitis workup was done in the setting of consistent pembrolizumab use although the patient did not report hypophysitis-related symptoms. Labs were notable for low insulin-like growth factor 1 (IGF-1), low total triiodothyronine (T3), and low luteinizing hormone (LH). Thyroid-stimulating hormone (TSH), free thyroxine (T4), adrenocorticotropic hormone (ACTH), cortisol, follicle-stimulating hormone (FSH), and anti-glutamic acid decarboxylase (anti-GAD) were within normal limits. Other autoantibodies implicated in type I diabetes mellitus (T1DM) such as islet cell antibodies (ICA) and insulin autoantibodies were not tested in the acute setting. The patient was transferred to the general medicine floor after anion gap closure. She was referred to an endocrinologist for outpatient management of ICI-related diabetes and was subsequently started on daily long-acting insulin of 10 units and meal-time insulin of five units. Pembrolizumab was discontinued by her oncologist. The patient remained on insulin for more than one year after discontinuation of pembrolizumab and will likely have a lifelong insulin requirement.

## Discussion

IRAEs associated with anti-PD1 agents are becoming more commonly reported. Systematic reviews and meta-analysis of these medications report fatigue and arthralgias as some of the most common side effects, with endocrinopathies rarely reported [4]. Specifically, pembrolizumab can cause mild adverse effects in up to 60% of patients and severe end-organ toxicities such as nephritis, colitis, and pneumonitis in <10% of patients [5]. Pembrolizumab-induced DKA was reported in only 0.1% of the patients enrolled in clinical trials [6].

We report a rare case of pembrolizumab-induced DKA in an elderly patient with no prior diagnosis of diabetes. The autoimmune workup did not reveal anti-GAD antibodies which are typically present in 80% of patients with T1DM. However, the presence of ICA and insulinoma-2-associated (IA-2A) autoantibodies at time of diagnosis for T1DM range from 69% to 90% and 54% to 75%, respectively, and were not tested in our patient in the acute care setting [7]. The specific pathophysiology of IRAEs remains unknown. Studies using murine models have implicated the role of both antibody-dependent cell-mediated cytotoxicity (ADCC) and complement pathway activation [8]. The involvement of autoimmune diabetes mellitus (DM) antibodies in the pathogenesis of ICI-related DM also remains unclear with varying data on the percentage of patients with positive antibodies.

The differences in clinical presentation between T1DM and ICI-related DM are important to recognize in the acute care setting. Fulminant T1DM presents with elevated pancreatic enzymes (amylase and lipase) in 98% of patients but was rarely seen in ICI-related DM [9]. Flu-like symptoms were also commonly reported in fulminant T1DM but uncommon in reports of ICI-related DM [8].

Most commonly, DKA in the critical care setting is triggered by infection (30%), a new diagnosis of diabetes in a young patient (25%), or non-adherence to medication (20%) [10]. It is important to consider ICIs as a cause of DKA, particularly in patients with a known cancer diagnosis. A detailed medication history is necessary and should include a review of past medications that have been discontinued as adverse events can persist long after discontinuation of a drug. The time of onset of IRAEs, specifically endocrinopathies, can vary from weeks to months from the initial dose of medication. While management of DKA in the critical care setting is largely unchanged, outpatient management with permanent insulin therapy is frequently needed, along with a complete autoimmune workup.

## Conclusions

IRAEs are becoming more commonly reported as more indications for immunotherapy are being discovered. The current data demonstrate that endocrinopathies are a rare side effect of ICIs. We have identified ICIs as a relatively novel precipitating factor of DKA. This study highlights similarities and differences in the presentation of DKA secondary to ICIs compared to the classical presentation of DKA secondary to type 1 diabetes mellitus. The majority of patients with DKA secondary to T1DM have presence of serum anti-GAD, anti-ICA, and anti-IA2A. The lack of these antibodies should raise concern for an IRAE in the appropriate setting. Lack of typical symptoms can also help distinguish DKA secondary to ICIs as our patient only presented with fatigue whereas many patients with DKA secondary to T1DM have numerous symptoms including polyuria, myalgia, nausea, and vomiting. This case adds to a nuanced understanding of DKA presentation and elucidates a rare side effect of immune checkpoint inhibitors.
